# The complete mitochondrial genome of *Trirachys orientalis* (Coleoptera: Cerambycidae)

**DOI:** 10.1080/23802359.2026.2642522

**Published:** 2026-03-11

**Authors:** Xiaolei Song, Guoqing Wan, Fengzhu Zhang

**Affiliations:** a Collaborative Innovation Center for Biomedicine, Shanghai University of Medicine and Health Sciences, Shanghai, China; b School of Pharmacy, Shanghai University of Medicine and Health Sciences, Shanghai, China; c Shanghai Yangpu District Mental Health Center, Shanghai University of Medicine and Health Sciences, Shanghai, China

**Keywords:** Cerambycinae, mitogenome, phylogenic relationship

## Abstract

*Trirachys orientalis* (Hope, 1841) is a long-horned beetle widely distributed in China with economic importance and potential medicinal value. In this study, we sequenced and assembled its complete mitochondrial genome. The complete mitogenome is 16,028 bp in length with a GC content of 29.60%, including 13 protein-coding genes, 2 ribosomal RNA genes, 22 transfer RNA genes, and a control region. We further made the phylogenetic tree on the complete mitochondrial genomes of other 10 closely related species to show their phylogenic relationship. This genomic resource provides a fundamental baseline for future species identification, phylogenetic resolution, and the exploration of its potential utility as a biological resource.

## 1. Introduction

Cerambycidae (long-horned beetles) is a highly diverse family in Coleoptera, comprising over 35,000 described species (Allison et al. 2004; Lee and Lee 2020; Kim and Farrell 2025). The subfamily Cerambycinae is the second largest, with over 11,000 species globally (Lee and Lee 2020). While often regarded as forestry pests, many cerambycids are explicitly recorded as medicinal resources in Traditional Chinese Medicine (Namba et al. 1988). Supporting these traditional applications, modern research continues to uncover their broader pharmacological potential (Deyrup et al. 2021).

However, the high species richness and morphological variability of Cerambycidae, combined with the concealed lifestyle of their larvae, have historically complicated their taxonomy and phylogenetic resolution (Jin et al. 2022). To address these uncertainties, mitogenomes are increasingly used as informative molecular markers due to their maternal inheritance and rapid evolutionary rate (Cameron 2014; Wang et al. 2019; Haddad et al. 2021; Nie et al. 2021). Despite the availability of mitogenomes for many insects, data for Cerambycidae remains disproportionate to its diversity (Timmermans et al. 2010).

*Trirachys orientalis* (Hope, 1841) is widely distributed in China. Given the expanding pharmacological research on related cerambycids (Zhong et al. 2025), *T. orientalis* represents an abundant bio-resource with significant potential. However, the lack of genomic data hinders accurate species identification and evolutionary analysis. In this study, we sequenced and assembled the complete mitogenome of *T. orientalis* and reconstructed its phylogenetic position. This aims to clarify its evolutionary status within the subfamily Cerambycinae and provide a fundamental genomic baseline for future phylogenetic studies.

## 2. Materials and methods

### 2.1. Sample collection and DNA extraction

**Figure 1.**
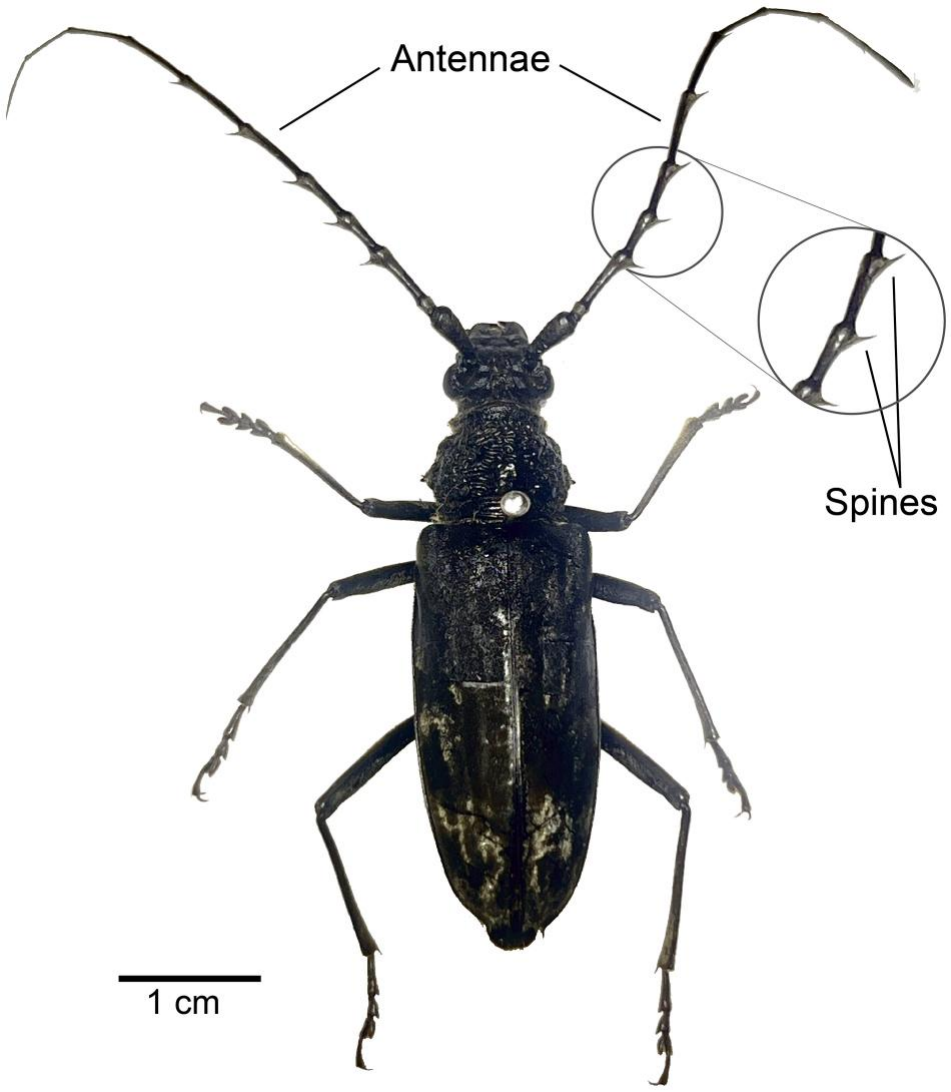
Adult habitus of Trirachys orientalis (Hope, 1841). The adult body length ranges from 35 to 52 mm, with a grayish-black or brownish-black integument densely covered in brownish-yellow and silver-gray silky pubescence. The antennae are longer than the body, distinct spines are present at apices of antennal segments. Photo taken by Xiaolei Song.

An adult female *Trirachys orientalis* was collected from Tushan, Bengbu, Anhui Province, China (32°55′57.3″N, 117°13′41.2″E) in July 2025. The specimen was preserved in 100% ethanol at −20 °C and deposited at Shanghai University of Medicine and Health Sciences (Voucher number: SUMHS020250726; Contact: Xiaolei Song, email: songxl@sumhs.edu.cn). The morphological characteristics of the adult specimen are shown in Figure 1. Total genomic DNA was extracted from thoracic muscle tissue of a single adult female (the voucher specimen) using the CTAB method (Reineke et al. 1998), fragmented to 350–500 bp, and sequenced on the Illumina NovaSeq 6000 platform. The raw sequencing data have been deposited in the NCBI Sequence Read Archive (SRA) under accession number SRR36576734.

### 2.2. Genome assembly and annotation

Raw data quality was evaluated using FastQC. A total of 30,297,158 reads (Q20 = 98.63%) were generated. High-quality reads were assembled *de novo* using GetOrganelle v1.7.7.1 (Jin et al. 2020) with optimized K-mer values. Annotation was performed using MitoZ v2.4 (Meng et al. 2019) and manually corrected in Geneious Prime 2022.2.2 (Kearse et al. 2012). The average sequencing depth was 1,452.5× (Supplementary Figure S1). The circular map of the mitochondrial genome was visualized using OGDRAW v1.3.1 (Lohse et al. 2007). The assembled complete mitogenome has been deposited in GenBank under accession number PX842584.

### 2.3. Phylogenetic analysis

Mitogenome sequences of eight closely related Coleoptera species and two outgroups were obtained from GenBank. The sequences were aligned using MAFFT v7.490 (Katoh and Standley 2013). A maximum-likelihood (ML) phylogenetic tree was constructed using FastTree v2.1.11 (Price et al. 2010) under the default GTR+CAT nucleotide substitution model, with branching reliability assessed *via* Shimodaira-Hasegawa (SH) test.

## 3. Results

### 3.1. Mitogenomic characteristics

**Figure 2.**
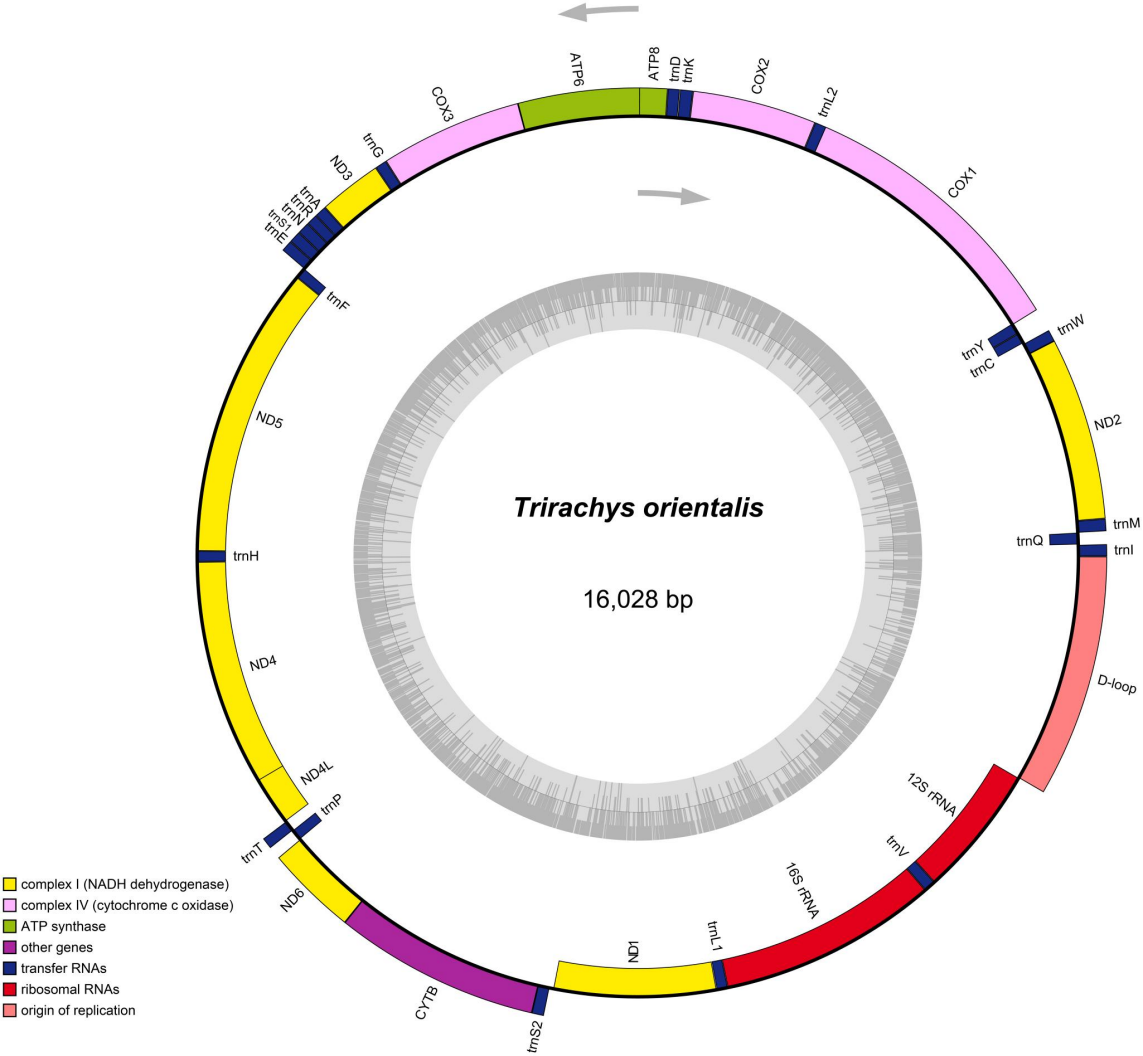
The sequence map of the mitochondrial genomes of Trirachys orientalis.

The complete mitochondrial genome of *Trirachys orientalis* (GenBank accession no. PX842584) was assembled as a closed, gap-free circular molecule of 16,028 bp (Figure 2). The nucleotide composition is 37.50% A, 32.90% T, 18.41% C, and 11.19% G, with a GC content of 29.60%, comparable to the related *Nadezhdiella cantori* (28%) (Tian and Li 2022). The mitogenome contains 37 genes, including 13 protein-coding genes (PCGs), 22 transfer RNA (tRNA) genes, 2 ribosomal RNA (rRNA) genes and 1 control region (D-loop). Fourteen genes (4 PCGs, 8 tRNAs, and 2 rRNAs) are encoded on the heavy strand (H-strand), while the remaining 23 genes (9 PCGs and 14 tRNAs) are located on the light strand (L-strand).

The 13 PCGs range in length from 156 bp to 1,720 bp (*ND5*). All PCGs initiate with ATN start codons (ATT or ATG), except *ND1* (TTG). Most PCGs (*ATP6*, *COX1*, *CYTB*, *ND2*, *ND4L*, and *ND6*) terminate with TAA, while *ND1* and *ATP8* use TAG. Five PCGs (*COX2*, *COX3*, *ND3*, *ND*4, and *ND5*) end with an incomplete stop codon (T–), a common feature completed by post-transcriptional polyadenylation (Kummer and Ban 2021). The 22 tRNA genes range from 63 bp (tRNA^Arg^) to 71 bp (tRNA^Lys^). Their GC content ranges from 16.92% (tRNA^Gly^ and tRNA^Glu^) to 43.28% (tRNA^Ile^). The 12S rRNA gene is 779 bp in length with a GC content of 27.98%, while the 16S rRNA gene is 1,289 bp long with a GC content of 24.05%. The control region (D-loop) spans 1,345 bp and is located between the 12S rRNA and tRNA^Ile^ genes.

### 3.2. Phylogenetic analysis

**Figure 3.**
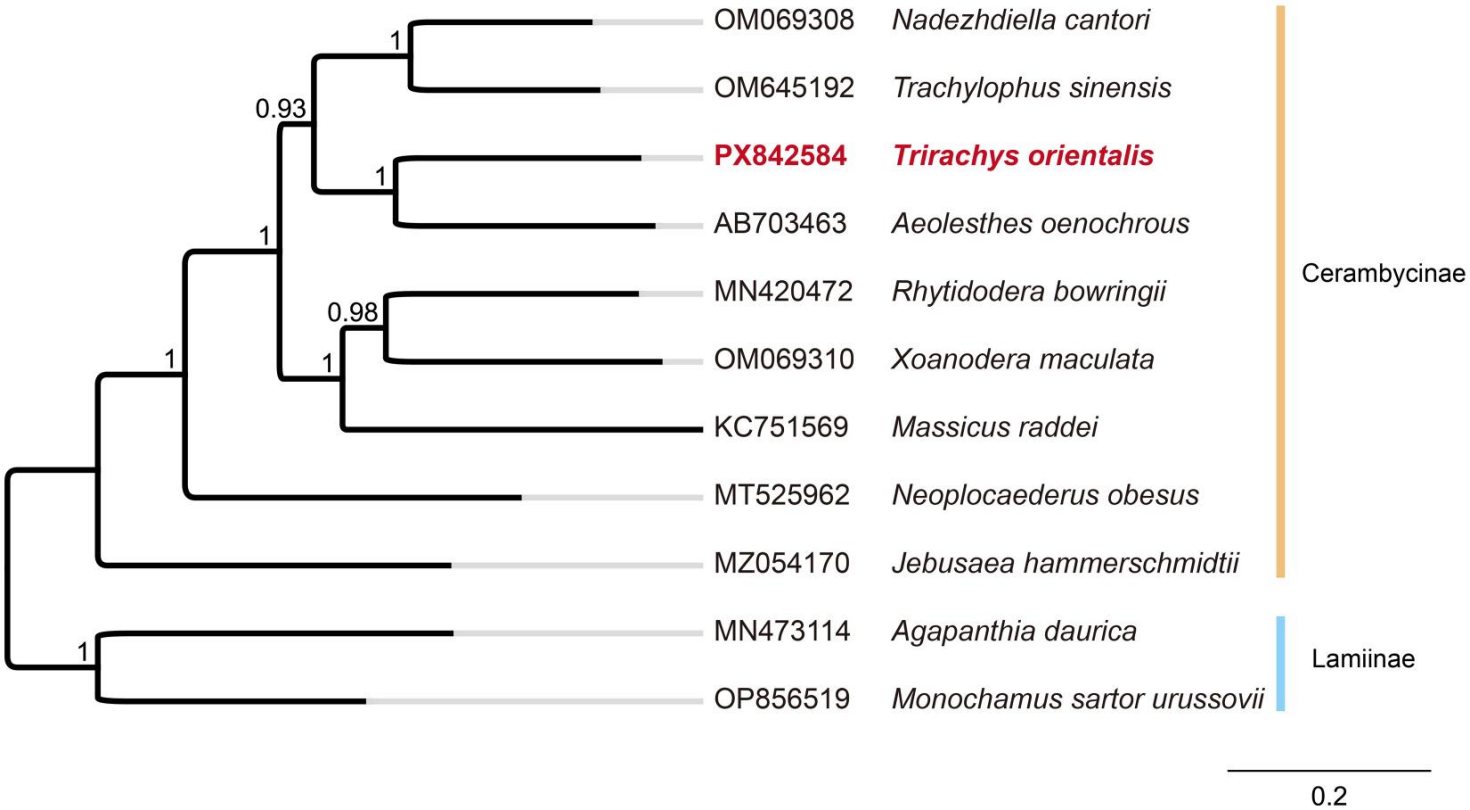
Maximum likelihood tree of complete mitochondrial genome of trirachys orientalis and 10 other closely species. Numbers on nodes are Shimodaira-Hasegawa (SH) test support values. The GenBank accession numbers and references for the sequences used in this analysis are as follows: Trirachys orientalis (PX842584, this study), nadezhdiella cantori (OM069308) (Tian and Li 2022), aeolesthes oenochrous (AB703463) (Chiu et al. 2016), massicus raddei (KC751569) (Wang et al. 2016), neoplocaederus obesus (MT525962) (Li and Lu 2020), trachylophus sinensis (OM645192) (Guo et al. 2022), xoanodera maculata (OM069310, unpublished), jebusaea hammerschmidtii (MZ054170) (Dias et al. 2021), rhytidodera bowringii (MN420472) (Nie et al. 2021), agapanthia daurica (MN473114) (Nie et al. 2021), and monochamus sartor urussovii (OP856519, unpublished).

The phylogenetic analysis included 11 species (Figure 3). The ingroup comprised nine species (Chiu et al. 2016; Wang et al. 2016; Li and Lu 2020; Dias et al. 2021; Nie et al. 2021; Guo et al. 2022; Tian and Li 2022) from the subfamily Cerambycinae, while two species (Nie et al. 2021) from the subfamily Lamiinae served as outgroups. Detailed accession numbers and references for all sequences are listed in the legend of Figure 3. The topology shows that the subfamily Cerambycinae is monophyletic, which is consistent with previous phylogenetic studies (Tian and Li 2022). The two Lamiinae outgroups formed a distinct clade separated from the nine Cerambycinae species. Within the Cerambycinae, *T. orientalis* was recovered as the sister group to *Aeolesthes oenochrous* with high node support values.

## 4. Discussion

In this study, we successfully characterized the complete mitochondrial genome of *Trirachys orientalis*. The structural features, including gene arrangement and high A + T content, align with patterns observed in ancestral insects and other long-horned beetles.

Phylogenetic reconstruction strongly supports the monophyly of the subfamily Cerambycinae, consistent with previous molecular studies (Tian and Li 2022). Within the tribe Cerambycini, *T. orientalis* formed a robust sister group with *Aeolesthes oenochrous*, clarifying its evolutionary position. The addition of this newly sequenced mitogenome enriches the genetic data available for Cerambycini, providing a reliable molecular basis for the evolutionary and taxonomic studies of this group.

Beyond taxonomy, this genomic resource provides a foundation for future exploration. While medicinal applications of several cerambycid species have been noted (Zhong et al. 2025), the biological properties of *T. orientalis* remain largely unexplored. Therefore, the complete mitogenome assembled here establishes a reliable genetic baseline not only for accurate species identification but also for future comparative genomics and pharmacological screenings to evaluate its potential economic and medicinal value.

## Supplementary Material

Sup_v2.docx

## Data Availability

The mitogenome sequence data supporting the findings of this study are openly accessible in GenBank at NCBI (https://www.ncbi.nlm.nih.gov) under accession number PX842584. The associated Bioproject, BioSample, and SRA numbers are PRJNA1392693, SAMN54270921 and SRR36576734, respectively.
