# Supplementary material for: The complete mitochondrial genome of Trirachys orientalis (Coleoptera: Cerambycidae)
Source: Mitochondrial DNA B Resour. 2026 Mar 11;11(4):531–5. doi: 10.1080/23802359.2026.2642522 (PMC12981255; doi:10.1080/23802359.2026.2642522)
Supplement: Sup_v2.docx [file TMDN_A_2642522_SM5688.docx]

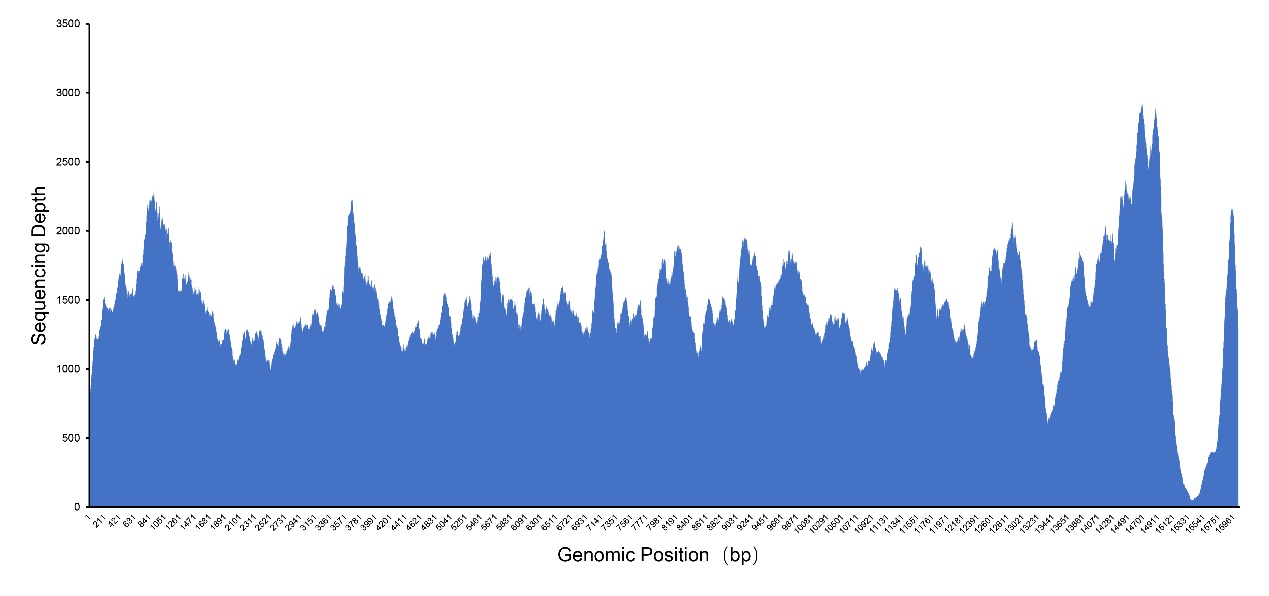
**Supplemental Material**

**Supplementary Figure S1. Coverage of the mitochondrial sequence of *Trirachys orientalis.*** The average sequencing depth was 1,452.5×.
